# Cell morphology and mechanosensing can be decoupled in fibrous microenvironments and identified using artificial neural networks

**DOI:** 10.1038/s41598-021-85276-5

**Published:** 2021-03-15

**Authors:** Edward D. Bonnevie, Beth G. Ashinsky, Bassil Dekky, Susan W. Volk, Harvey E. Smith, Robert L. Mauck

**Affiliations:** 1 McKay Orthopaedic Research Laboratory, Orthopaedic Surgery, University of Pennsylvania, Philadelphia, USA; 2 Translational Musculoskeletal Research Center, CMC VA Medical Center, Philadelphia, USA; 3 Department of Biomedical Engineering, Drexel University, Philadelphia, USA; 4 Department of Clinical Sciences and Advanced Medicine, School of Veterinary Medicine, University of Pennsylvania, Philadelphia, USA; 5 Department of Bioengineering, University of Pennsylvania, Philadelphia, USA

**Keywords:** Mechanotransduction, Cellular imaging

## Abstract

Cells interpret cues from and interact with fibrous microenvironments through the body based on the mechanics and organization of these environments and the phenotypic state of the cell. This in turn regulates mechanoactive pathways, such as the localization of mechanosensitive factors. Here, we leverage the microscale heterogeneity inherent to engineered fiber microenvironments to produce a large morphologic data set, across multiple cells types, while simultaneously measuring mechanobiological response (YAP/TAZ nuclear localization) at the single cell level. This dataset describing a large dynamic range of cell morphologies and responses was coupled with a machine learning approach to predict the mechanobiological state of individual cells from multiple lineages. We also noted that certain cells (e.g., invasive cancer cells) or biochemical perturbations (e.g., modulating contractility) can limit the predictability of cells in a universal context. Leveraging this finding, we developed further models that incorporate biochemical cues for single cell prediction or identify individual cells that do not follow the established rules. The models developed here provide a tool for connecting cell morphology and signaling, incorporating biochemical cues in predictive models, and identifying aberrant cell behavior at the single cell level.

## Introduction

Fibrous microstructural elements comprising the extracellular matrix (ECM) of most tissues convey critical biophysical and biomechanical cues to resident cells. Cells continuously interrogate this local microenvironment via contractile cytoskeletal proteins, and this information is relayed to the nucleus via the mobilization of mechanosensitive factors^1–3^. For many cells, these mechanical signals dictate their subsequent behaviors, such as differentiation (e.g., progenitor/stem cells) and/or activation (e.g., myofibroblasts)^4^. For many cells, cell–matrix feedback is mediated by Rho/ROCK and acto-mysosin based contractility. In such circumstances, mechanical properties of the cellular environment tunes this contractile behavior, where stiff microenvironments promote the emergence of pro-fibrotic phenotypes through increased contractile activity^5,6^, and soft microenvironments do the reverse.

While these findings hold in general, cell-to-cell variation is evident when populations are assessed at the single cell level. For example, we recently showed that, in unconstrained two-dimensional culture, sister cells that had recently divided take on markedly different morphologies and RNA copy numbers within a few hours of division^7^. This cell-to cell-variation within a population can be attenuated or amplified by imposing boundary constraints or contact guidance cues^8,9^. For example, cells on small adhesive islands have a lower overall cytoskeletal contractility, a regular geometry, and reduced activity of mechanosensitive factors, such as YAP/TAZ (Yes-associated protein/transcriptional coactivator with PDZ binding motif)^10^. Conversely, when boundaries are not constrained (e.g., in two-dimensional (2D) culture) cells adopt a variety of cell shapes and sizes. In this context, natural variations in cell shape and activity result in significant variation in cellular behavior, and can promote changes in fate and activity of a subpopulation of the cells, such as invasive behavior^11–15^. This natural variation can be amplified as well. For example, in fibrous networks, increases in structural variation in disorganized compared to aligned fiber microenvironments drive cell mechanosensing^2^. These data indicate that physical factors and microenvironmental cues are integrated through cellular processes to dictate cell function. Further, conditions that enable or promote cell-to-cell variation can accentuate heterogeneous cell behaviors within an otherwise homogeneous population.

As noted above, when cells integrate cues from their microenvironment, they adopt a wide range of shapes, sizes, and orientations^1,2,14^, and a number of descriptors or metrics are reported (e.g., area, roundness, aspect ratio) to describe this phenomenon. These include both descriptors of the cell body as well as the nucleus, yielding complex, multidimensional data sets, where multiple measures have weak correlations with functional outcomes. To better integrate these multiple inputs, machine learning can translate and simplify these data^15–18^, and dimensional reduction, clustering, predicting, and classifying can reveal how populations of cells integrate microenvironmental cues to dictate function.

Here, we utilize machine learning approaches to establish how cells in heterogeneous microenvironments regulate mechanosensing on a single cell basis. To do so, we fabricated fibrous biomaterials with tunable organization based on stretch-mediated fiber organization. Cell morphology was heterogeneous in these environments, but dimensional reduction using a neural network-generated self-organizing map identified subsets of cell shapes by identifying four clusters of cell and nuclear morphology. Quite interestingly, these clusters represented different mechanobiological states (i.e., YAP/TAZ localization), and such states were predictable on an individual cell basis. The model further identified atypical cellular behaviors, when morphology and mechanobiologic state were decoupled (for example by pharmacologically altering cellular contractility). Moreover, the model could identify cases where the morpho-mechanobiologic relationship changed, during developmental specification of lineage (in skin cells) and in disease (transformation to an invasive phenotype in cancer cells).

## Results

### Strain-mediated reorganization and contact guidance in fibrous microenvironments

Many tissues exist in a prestressed state (e.g., skin, tendon, ligament, and the annulus fibrosus of the intervertebral disc)^19–21^. We recently reported that loss of this prestressed state, associated with disease or injury, can alter fiber organization in these tissues. This disorder in the fiber topography instigates the emergence of atypical phenotypes, such as pro-fibrotic, α-smooth muscle actin (αSMA) positive cells^2^. As topography is tied to the state of strain in a fiber environment^22,23^, we first explored how organization relates to the state of stretch in these networks. To do this, we developed a fiber mechanics model to include a state variable governing fiber organization^24^. The development of the model is described in detail in the methods section, and results in the expression:

1 $$S_{11} = \mathop \smallint \limits_{0}^{{\varepsilon_{11} }} \mathop \smallint \limits_{ - \pi /2}^{\pi /2} R\left( {\theta ,\varepsilon } \right) \cos^{2} \theta A B \exp \left( {B\varepsilon } \right) d\theta d\varepsilon + k_{matrix} \varepsilon_{11.}$$

where the axial stress (*S*_11_) is described by the strain-mediated (*ε*) fiber organization (*R*) and material constant fitting parameters. We tested this model on aligned and non-aligned electrospun scaffolds that were subjected to different levels of strain (Figs. S1, S2) and found that for low strain (within the toe region), the model fit exceptionally well (R^2^ > 0.9) for both the aligned and non-aligned networks (Fig. S1).

Given the relationship between fiber topography and state of strain, we next assessed how this fiber reorganization regulates the morphological state of cells that typically reside in a highly aligned, prestressed fiber environment. For this, bovine annulus fibrosus cells (bAFC) were seeded on aligned and non-aligned fiber networks that were stretched to 0, 3, 6, or 9% strain prior to cell seeding. Fluorescent imaging the actin cytoskeleton and nucleus revealed that cell spreading was heterogeneous (Fig. S1), but that cell and nuclear shape descriptors generally had positive or negative correlations with fiber organization (Fig. S3). Notably, many descriptors in this multidimensional data set were statistically inter-related to one another (Fig. S3), making direct predictors of how topography dictates cell shape and size difficult to identify without a more sophisticated method to analyze the multidimensional data.

### Unsupervised clustering of morphologic states in fibrous microenvironments

**Figure 1:**
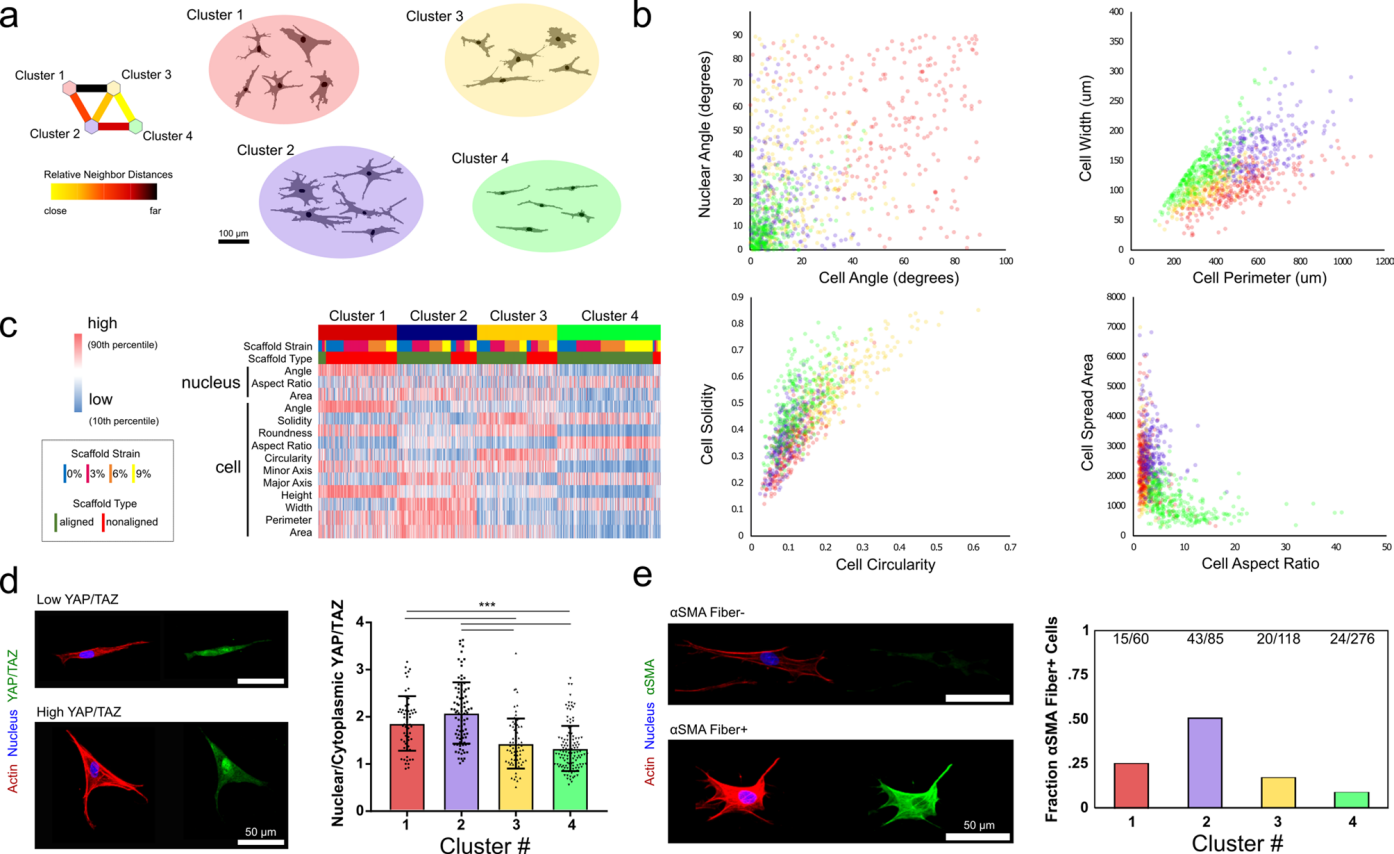
Dimensionality reduction predicts relationship between cell morphology and mechanosensing in engineered fiber environments. (**a**) Images of cells were segmented and 14 cell and nuclear morphology parameters were collected for each. These data were simplified via a neural-network-based self-organizing map approach to dimensional reduction that identified four shape groups. (**b**) The distinction between groups is highlighted by relationships between cell and nuclear descriptors of size, shape, and orientation. (**c**) Shape clusters were related to both baseline fiber organization and degree of stretch-mediated reorganization. (**d**) YAP/TAZ imaging on a subset of cells revealed a connection between shape clusters and mechano-sensing, as indicated by nuclear/cytoplasmic levels of YAP/TAZ. (**e**) These same clusters also represented different propensities of adopting a pro-fibrotic α-smooth muscle actin + phenotype (numbers denote number of cells identified as being αSMA stress fiber + over total cells identified in that cluster). (*** denotes p < 0.001, n = 1043 cells for initial cluster analysis, n = 338 cells for YAP, number for αSMA cells given above bars as positive over total cells per cluster).

Due to the complexity of this 14-dimensional data set, we turned to a neural network approach for dimensionality reduction using an unsupervised learning algorithm. To do this, we developed a self-organizing map neural network composed of 2 neurons that classifies cells onto a 2 × 2 grid via a competitive learning algorithm (Fig. 1a)^25^. This 2 × 2 map identified 4 groups of morphologies that are distinguishable by eye (Fig. 1a). Generally, the 14 morphology parameters of cell and nuclear descriptors distinguished these clusters, and comparing several of these metrics highlighted the differences between the groups (Fig. 1b). Notable distinctions occurred between the clusters: group 1 represented cells spreading off the fiber axis with low solidity and low aspect ratios, group 2 represented cells with high area, high perimeter, and low circularity, group 3 represented cells with high circularity and low nuclear aspect ratio, and group 4 represented cells with low area and high aspect ratio (Fig. 1b,c). While many studies modulate cell shape and size through surface modifications of 2D substrates^8–10^, the heterogeneity of these fiber environments engendered considerable cell-to-cell shape variations. As expected^2,26,27^, there was a strong connection between the fiber organization and cell shape, both in terms of the baseline fiber organization and degree of strain on the fiber network (Fig. 1c, Fig. S4).

Because morphology and mechanically-driven phenotypes are linked in many cell types, we next tested whether the shape clusters identified via the neural network represented different mechanobiologic states. Individual cells were imaged in a subset of the fiber environments (aligned and nonaligned scaffolds at 0% and 9% strain) for both cell and nuclear morphology and the localization of the transcription factors YAP/TAZ. These data showed pronounced differences in nuclear YAP/TAZ levels between shape clusters (Fig. 1d). To determine whether these shape clusters similarly predicted shifts in phenotype, we stained for αsmooth muscle actin (αSMA), which is indicative of a pro-fibrotic phenotype^28^. Results from this analysis showed that shape clusters indeed identified different propensities toward this pro-fibrotic phenotype (Fig. 1e). These data indicate that neural networks may have the capacity to identify subsets of cells that have differing mechanobiological states and phenotypes within a heterogeneous population, based on cell and nuclear morphology.

### Neural network modeling using cell and nuclear morphology predicts mechanobiologic state

**Figure 2:**
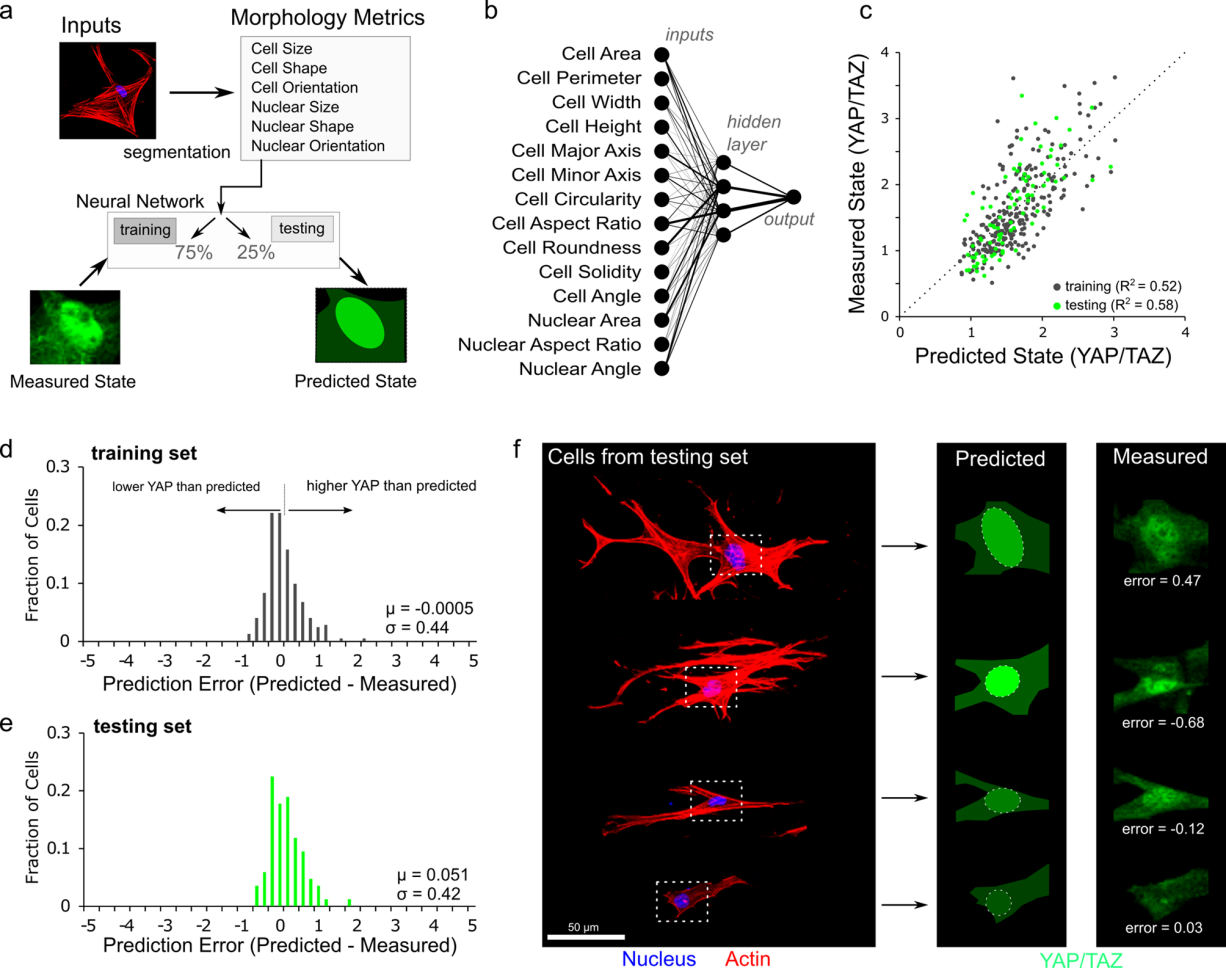
A neural network uses cell morphology to predict mechanobiologic state. (**a**) A neural network was constructed by segmentation of individual cells to quantify cell and nuclear size, shape, and orientation and trained by Bayesian regularization based on measured YAP/TAZ levels. (**b**) The neural network consisted of 14 inputs and a 4 neuron hidden layer to predict the nuclear/cytoplasmic ratio of YAP/TAZ. (**c**) Both training and testing data sets maintained predictability with R^2^ between predicted and measured states above 0.5. Error histograms for training (**d**) and testing (**e**) sets were centered close to zero error (µ_train_ = − 0.0005 and µ_test_ = 0.051) with associated standard deviations of σ_train_ = 0.44 and σ_test_ = 0.42. (**f**) Predicted values for cells from the testing set provide a visual depiction of different error values.

Given the above connection between cell and nuclear morphology and mechanosensing, we next questioned whether mechanobiologic state could be predicted for single cells based solely on cell and nuclear shape descriptors. To accomplish this, we developed a supervised neural network of 4 hidden-layer neurons with sigmoid transfer functions trained through Bayesian regularization (Fig. 2a). In the model, nuclear/cytoplasmic levels of YAP/TAZ were predicted based on the shape descriptors described above (Fig. 1). Using a 75%–25% data split for training and testing, we found that greater than 50% of the variation in YAP/TAZ localization in single cells could be predicted by these shape descriptors (Fig. 2b,c). Consistent with previous studies^8,10^, the most important predictive parameters in the model were cell aspect ratio, cell area, and nuclear area (Fig. 2b, Fig. S5).

Our engineered fiber environments generate a large dynamic range in cell and nuclear morphology (Fig. S1) and this provides a robust data set for models relating morphometry and mechanosensing. We further assessed the ability of this model to predict YAP/TAZ levels by inspecting the error histograms for cells in both training and testing sets (Fig. 2d,e; Fig. S6). In this analysis, the mean error (μ) describes whether or not the model systematically over (μ < 0) or under (μ > 0) predicts YAP/TAZ levels, and the standard deviation (σ) of the errors describes precision of the predictions. Further, these errors can be visualized by constructing prediction images using vector graphic software (Fig. 2f) to compare predictions and measured values. This error analysis and visualization supports that the model can predict localization of mechanoactive transcription factors.

To evaluate how generalizable this approach was, we next evaluated cells in the simpler microenvironments of 2D substrates of differing stiffness (5 kPa and 55 kPa polyacrylamide, and glass). In this context, the model again provided good agreement with predicted YAP/TAZ levels compared to measured values (Fig. S7). Here, we do not present YAP/TAZ levels qualitatively as nuclear or cytoplasmic, but as a continuous and quantitative metric that encompasses low, medium, and high activation states. Thus, to support the model predictions of mechanobiologic state, we used the model to compare functional outcomes of cellular traction forces and nascent matrix deposition at the single cell level with the predicted YAP/TAZ levels. For this, annulus fibrosus cells were seeded on an intermediate substrate stiffness (10 kPa polyacrylamide) and shape parameters were extracted via phase contrast imaging, followed by post hoc analysis of traction forces and prediction of YAP/TAZ localization for individual cells. These predicted values from the model were compared against contractile force (Fig. S8), with a strong correlation between predicted YAP/TAZ and total contractile force (R^2^ = 0.66) Additionally, single cell matrix deposition was measured as previously reported^2^ via functional non-canonical amino acid tagging and compared to predicted YAP/TAZ levels (Fig. S9). Again, this single cell functional outcome of YAP/TAZ activation correlated with the predicted levels (R^2^ = 0.422) providing insight to the spectrum of YAP/TAZ activation levels.

The original model formulation was established in a single cell type (bovine annulus fibrosus cells, AFCs), and consequently, we were curious to see whether the morphology-mechanobiology relationship that was determined for those cells could be applied more generally to other cell types. To address this, we evaluated bovine marrow-derived mesenchymal stromal cells (bMSCs) in both organized and disorganized fiber environments. Testing this data set in the established neural network developed based on annulus fibrosus cells showed similar accuracy between MSCs and the AFCs (Figs. S6 and S10). Thus, this morphology-mechanobiology relationship held across mesenchymal lineage cells. However, it remained unclear as to whether this model captures a universal rule set for cell spreading in general.

### Incorporation of biochemical cues can restore predictive capacity

**Figure 3:**
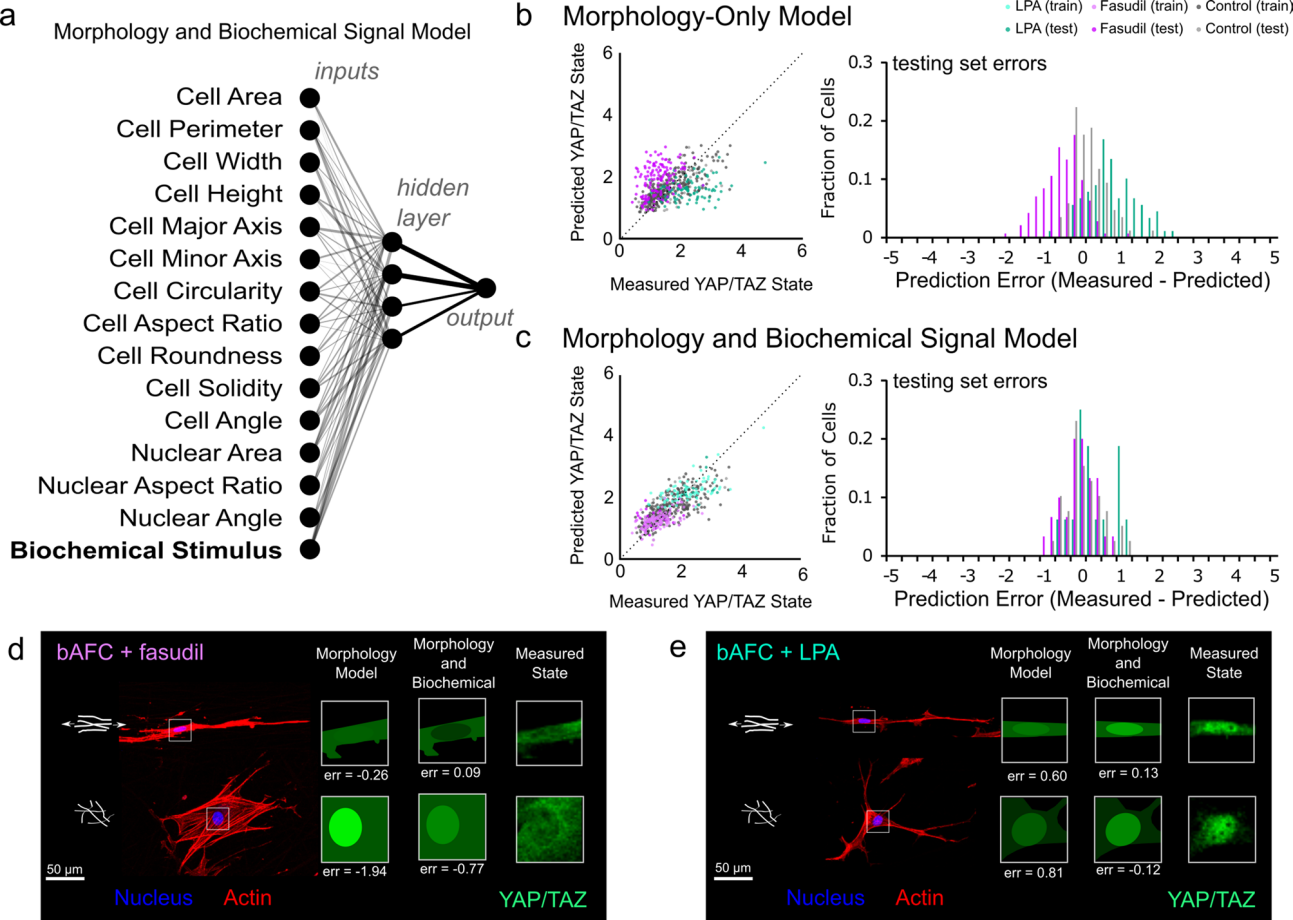
(**a**) To account for the role that biochemical signaling can play in YAP/TAZ localization, a neural network was constructed that incorporates cell and nuclear morphology in addition to information on biochemical signaling. Line thicknesses correspond to connection weights. (**b**) Using a morphology-only neural network provides either over (fasudil, ROCK inhibition) or under (LPA, rhoA activation) predictions of YAP/TAZ state. (**c**) Incorporating biochemical signaling into the model increases model accuracy with no loss of predictability for control group cells. Example cells from the (**d**) Fasudil and (**e**) LPA groups along with visual depictions of the model predictions for both models (new model: n_LPA-train_ = 73, n_LPA-test_ = 16, n_Fasudil-train_ = 112, n_Fasudil-test_ = 30, n_control-train_ = 299, n_control-test_ = 39 cells).

The model above proved robust for capturing cells following established ‘rules’ between spreading and mechano-response. This motivated us to ask whether and when this relationship might be disrupted. AFCs were cultured on organized and disorganized fiber environments and their cytoskeletal contractility was manipulated via ROCK inhibition (with fasudil) and RhoA activation (with lysophosphatidic acid (LPA)). In general, YAP/TAZ nuclear localization decreased with reduced contractility and increased with higher contractility (Fig. S11). While slight variations in cell spreading and shape accompanied these shifts, they did not account for the changes in YAP/TAZ (Fig. S6). Because of this disconnect, nuclear YAP/TAZ levels were overestimated following fasudil treatment (μ_fasudil_original_ = − 0.60, σ _Fasudil_original_ = 0.50) and underestimated following LPA treatment (μ_LPA_original_ = 0.69, σ _LPA_original_ = 0.64) using the model developed above (Fig. 3b, Fig. S12). As could be expected, this observation highlights that biochemical signaling can be a potent mediator of the model linking morphology and mechanosensing. Consequently, we aimed to develop a new model that is capable of incorporating this information to more accurately predict mechanobiologic state by incorporating biochemical cues as a model input (Fig. 3a). In this model, 15 inputs were fed through a 4 neuron hidden layer to predict state based on morphology and knowledge of contractility modulation. Incorporating this input data significantly enhanced predictive power by reducing the errors associated with independent testing of cells treated with either Fasudil (μ_fasudil_adapted_ = 0.01, σ _Fasudil_adapted_ = 0.35) or LPA (μ_LPA_adapted_ = − 0.01, σ _LPA_adapted_ = 0.51) with no loss of predictive power for cells in control media conditions (Fig. 3c,d,e; Fig. S12). The adaptation of a morphology-mechanosensing model to incorporate biochemical signals highlights the utility of harnessing the ability of neural networks to translate complex, multidimensional input data into a single quantitative output. Additionally, the accuracy of this model also supports that such models may be used to identify when cells, either individually or as a population, may deviate from the expected cell behavior.

### Neural networks to identify distinctly contractile cell populations

**Figure 4:**
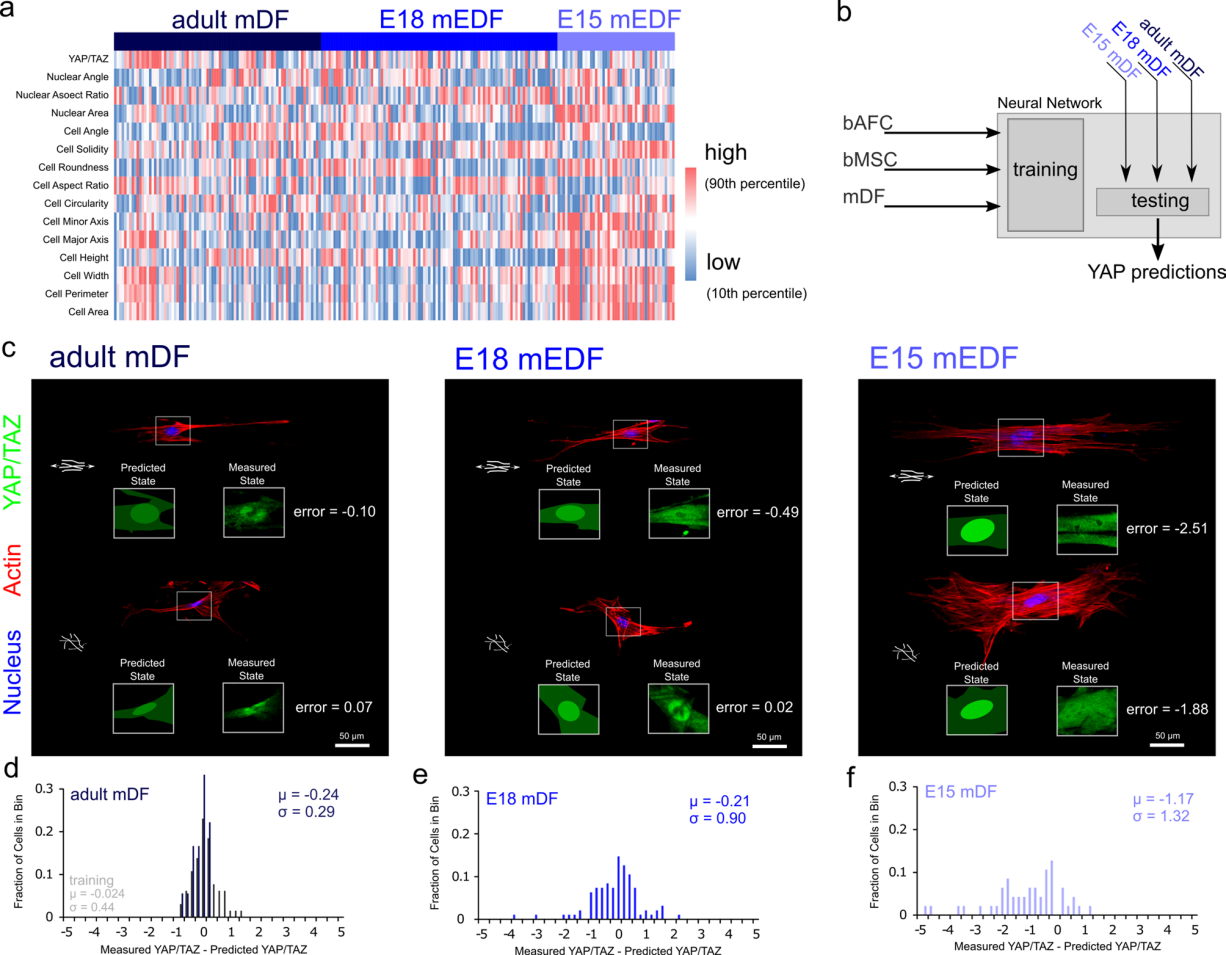
Developing murine dermal fibroblasts have emergent morphology-mechanosensing relationships. Murine dermal fibroblasts from adults and embryonic days 15 and 18 were isolated and seeded onto organized and disorganized fiber environments. (**a**) Staining for actin, the nucleus, and YAP/TAZ revealed heterogeneity and developmental stage specific morphology and mechanosensing. (**b**) The neural network described above (Fig. 3) was updated and retrained based on AFCs, MSCs, and adult murine dermal fibroblasts (mDFs) and tested on the remaining dermal fibroblast groups (**c**). In general, YAP/TAZ levels were predictable for (**d**) adult and (**e**) E18 cells (µ_adult_ = − 0.24, σ_adult_ = 0.29; µ_e18_ = − 0.21, σ_e18_ = 0.90). (f) However, YAP/TAZ levels were largely overestimated for dermal fibroblasts isolated from the mid-gestational fetus (E15), which is a stage associated with scarless healing (µ_e15_ = − 1.17, σ_e15_ = 1.32), (n_DF-train_ = 65, n_DF-test_ = 18, n_E18_ = 95, and n_E15_ = 47 cells).

With this in mind, we next examined a case where altered contractility is expected in cell populations, namely, embryonic wound healing^29,30^. Mid-gestational murine dermal wounds heal in a scarless fashion (prior to E16.5 in the mouse)^31,32^, with embryonic murine dermal fibroblasts (mDFs) maintaining lower contractility when isolated from scar-less healing (i.e., E15) compared to scar-forming (i.e., E18) stage embryos^33^. This is in agreement with the concept of highly contractile, mechanosensitive myofibroblasts as key mediators of scarring^28^. Thus, we next queried whether embryonic dermal cells alter their morphology-mechanosensing rules with development. To do so, adult, mid-gestational, and late-gestational embryonic (E15 and E18, respectively) dermal fibroblasts were isolated from mice, and cultured in fibrous environments. Both morphology and YAP/TAZ levels differed in cells from these different stages in development (Fig. 4a). We then expanded the neural network above by further including ground truth data of mesenchymal cells (bMSCs) and adult dermal fibroblasts (adult mDFs) (Fig. 4b, Fig. S6). This updated model showed robust predictability of adult (µ_adult_ = − 0.24, σ_adult_ = 0.29) and late-gestational (E18) mDFs (µ_e18_ = − 0.21, σ_e18_ = 0.90). In contrast, mid-gestational embryonic mDFs (E15) diverged from this model (µ_e15_ = − 1.17, σ_e15_ = 1.32), with YAP/TAZ levels considerably overestimated on the single cell level (µ_e15_ <  < 0). These cells, which are associated with low contractility and scarless healing (E15), were decoupled in their morphology and mechanosensing, and the model found significantly increased prediction errors for these cells compared to adult and E18 cells (Fig. S6). These data confirm that, for certain cell populations a morphology-mechanosensing neural network can identify conditions when cells or populations of cells do not act as expected.

### Neural network modeling of morphology and mechanobiology can identify invasive phenotypes in breast cancer cells

**Figure 5:**
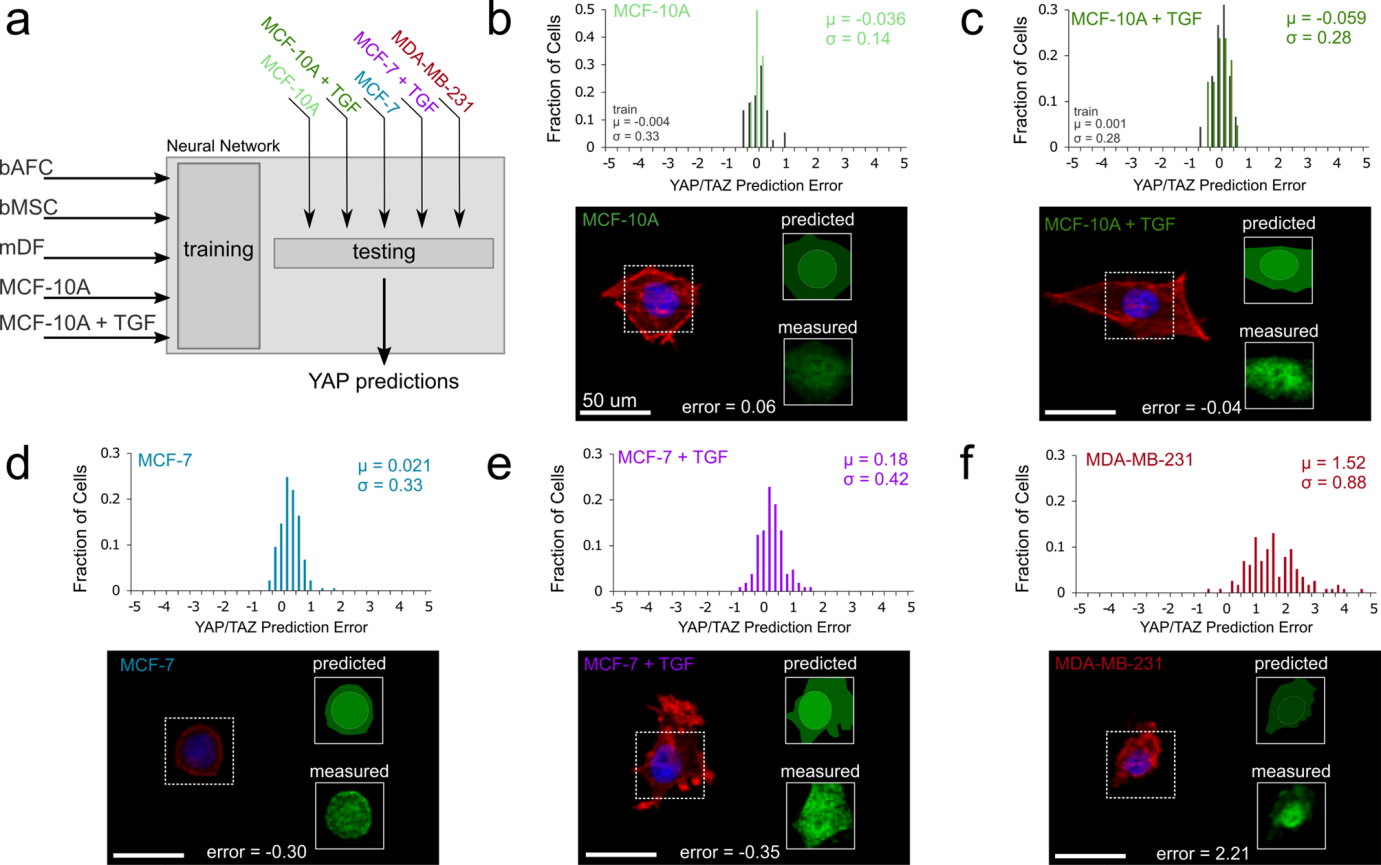
Non-tumorigenic breast epithelial and non-invasive breast cancer cells are predictable, while invasive breast cancer cells are not. (**a**) The neural network model was updated to include ground truth data on epithelial cells with and without TGF-β_1_ to instigate epithelial-to-mesenchymal transition (EMT). (**b**,**c**) Non-tumorigenic breast epithelial (MCF-10A) and (d,e) non-invasive breast cancer (MCF-7) cells showed predictable morphology-mechanosensing relationships, and this predictability remained when these cells were pushed towards EMT using TGF-β_1_. (f) In contrast, invasive breast cancer cells (MDA-MB-231) showed unpredictable YAP/TAZ levels based on cell and nuclear morphology (n_MCF-10A-train_ = 37, n_MCF-10A-test_ = 12, n_MCF-10A+TGF-train_ = 45, n_MCF-10A+TGF-test_ = 21, n_MCF-7_ = 177, n_MCF-7+TGF_ = 105, and n_MDA-MB-231_ = 115 cells).

Machine learning techniques to both understand and identify invasive cancer cells have emerged over the past several years^15,16,18^. While invasive cancer cells are generally more contractile than non-invasive cancer cells^34^, recent evidence also suggests that progression (i.e., proliferation) and invasion represent different mechanobiological states^35^. Specifically, YAP-mediated Hippo pathway activation is not present in progression, but downstream Hippo pathway targets are upregulated in invasive phenotypes^35^. Given that the neural network developed to predict YAP/TAZ localization can identify disconnects between morphology and mechanosensing, we next tested whether this approach could likewise identify the differential mechanobiology associated with cancer progression and invasion. To do so, we first exposed non-tumorigenic mammary epithelial cells (MCF-10A) to disorganized fiber environments with and without 10 ng/mL TGF-β_1_ to instigate epithelial to mesenchymal transition (EMT)^36,37^; this ground truth data was then incorporated into the morphology-only neural network described above (Fig. 5a). With this retrained model, we found high predictability both prior to (µ_10A_ = − 0.036, σ_10A_ = 0.14) and following the initiation of EMT (µ_10A+TGF_ = − 0.059, σ_10A+TGF_ = 0.28) (Fig. 5b,c; Fig. S6). We then tested whether non-invasive (MCF-7) and invasive (MDA-MB-231) breast cancer cells also act in a predictable manner. Non-invasive cancer cells were well predicted both in the presence and absence of TGF- β1 (μ_MCF7_ = 0.021, σ_MCF7_ = 0.33; μ_MCF7+TGF_ = 0.18, σ_MCF7+TGF_ = 0.42; Fig. 5d,e; Fig. S6). In contrast, the highly metastatic, invasive cancer cell line (MDA-MB-231) deviated markedly from model predictions, with YAP levels being substantially higher than expected based on cell and nuclear morphology (μ_231_ = 1.52, σ_231_ = 0.88, Fig. 5f, Fig. S6). These data suggest a disconnect between matrix mechanosensing and cell morphology during cancer cell invasion.

**Figure 6:**
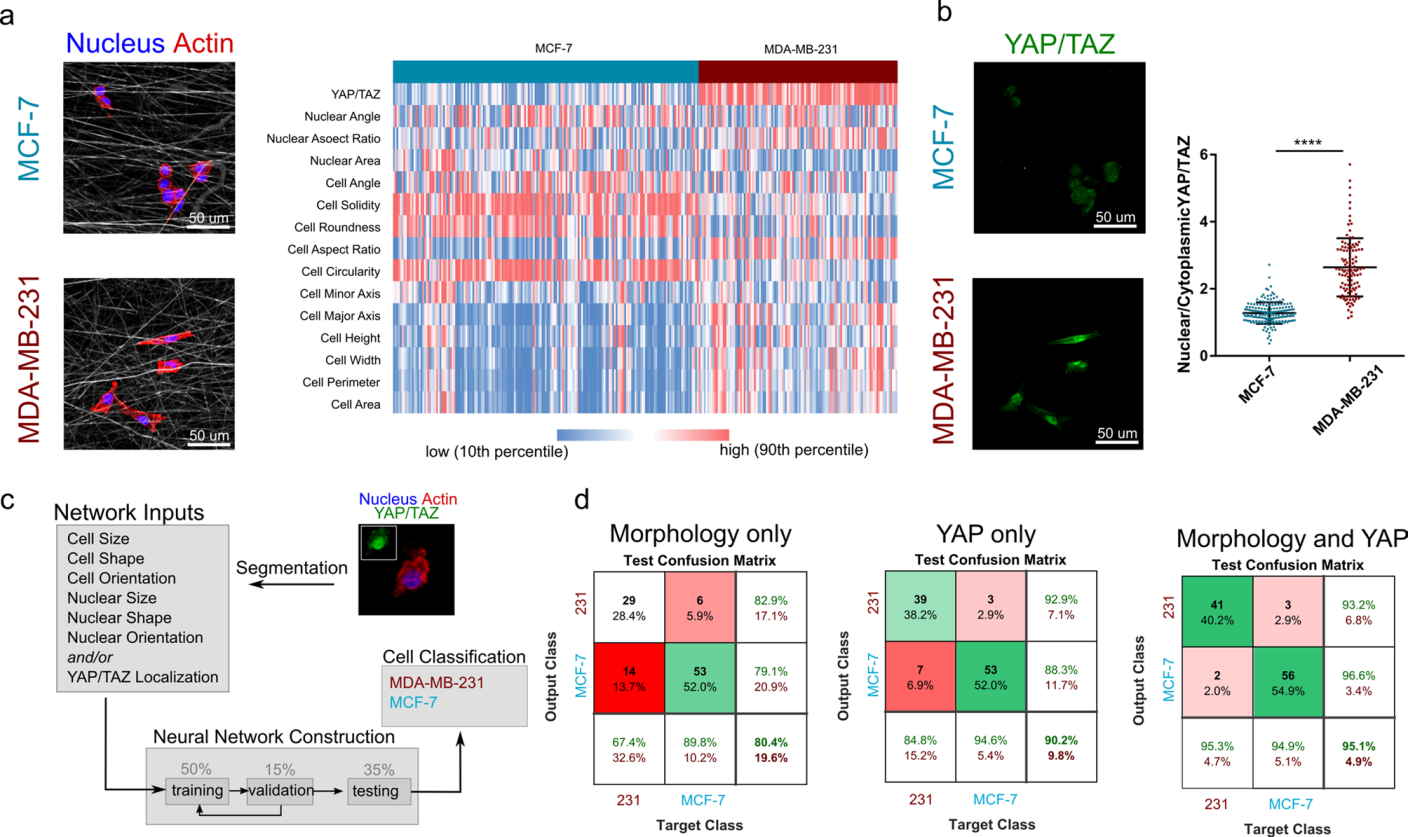
(**a**) Non-invasive and invasive cancer cells showed different, yet heterogeneous, morphologies on non-aligned fibrous templates, and this difference was highlighted by (**b**) different YAP/TAZ states. (**c**) A neural network was constructed to classify cells as invasive or non-invasive based on morphology and/or YAP/TAZ levels. (**d**) Using morphology to classify cells resulted in 80.4% accuracy, while YAP/TAZ predicted cell classification with 90.2% accuracy. Using both morphology and mechanosensing increased accuracy to 95.1%, highlighting the utility of exploiting both morphology and mechanosensing in invasive cancer cells (**** p < 0.001, n_MCF-7_ = 177 and n_MDA-MB-231_ = 115 cells).

Based on the above observation, we developed another neural network that was tasked with sorting cells into either the non-invasive or invasive class (MCF-7 versus MDA-MB-231). This sorting was based on cell and nuclear morphology, YAP/TAZ state, or a combination of both morphology and mechanobiological state. While it is clear that the non-invasive and invasive cancer cells adopt differing morphologies (Fig. 6a), there is also a significant difference in matrix mechanosensing via YAP/TAZ (Fig. 6b). To determine whether cell morphology or mechanosensing alone are sufficiently robust predictors of cancer invasiveness, we first developed neural networks to classify cells based solely on these parameters. Using morphology alone, the neural network maintained 80% accuracy in classifying cells as either invasive or not (Fig. 6c,d). However, testing this model on cells not included in the training step revealed a 14% false negative rate. Using YAP/TAZ levels alone for classifying invasiveness increased the overall accuracy to 90%, with a lower false negative rate of 6.9% (Fig. 6d). Notably, a third model that incorporated both morphology and YAP/TAZ (exploiting the morphology-mechanosensing disconnect identified, Fig. 5f) increased the accuracy of the model to 95% and reduced the false negative rate to 2% (Fig. 6d). Of note, the utility of fibrous networks in modulating mechanosensing and increasing the dynamic range in cell responses was further evidenced in this study. Recent evidence suggests that even soft 2D substrates can activate the Hippo pathway in MCF-7 cells while this YAP/TAZ-activated pathway remains inactive in 3D environments^35^. Thus, when these same cells were seeded onto 2D glass substrates, the model predictions were significantly worse, with morphology alone at 87%, YAP/TAZ alone at 63%, and their combination at 86% accuracy (Fig. S13). Thus, the difference in mechano-signaling between the invasive and non-invasive cancer cells was lost in the stiff 2D environment, highlighting the importance of the physiological microenvironment in regulating and accentuating cell behavior and classification.

## Discussion

As cells integrate microenvironmental cues through their contractile machinery, mechanosensitive transcription factors such as YAP/TAZ dictate downstream cellular responses. In this study we revealed that microenvironment-mediated cell spreading can predict the localization of these factors for a variety of cell types, including differentiated fibroblasts (bAFCs and mDFs), mesenchymal stromal/progenitor cells (bMSCs), and human mammary epithelial cells (e.g., MCF-10A). Despite differences in baseline phenotype between these cells, there was a notable conservation of the rules that govern how they translate cell spreading to mechanoactivation. While the relationship between cell spreading and contractility has been previously explored^2,10,38,39^, the power of the present model resides in its ability to identify time-varying and/or aberrant cell behavior. It is also important to note that these rule sets are not universal for all cells at all times. For instance, pharmacologically altering contractility shifted the model predictions in a manner where decreased contractility decreased YAP/TAZ nuclear levels in an unpredictable manner, and increasing contractility caused the opposite to occur. As such, another powerful use of such a model is to incorporate biochemical cue data into the input data set to account for this disconnect. In a more natural deviation from the expected cell behavior, mid-gestational (E15) mDFs and invasive breast cancer cells (MDA-MB-231) exhibited mechano-signaling that was decoupled from cell spreading. Thus, while this model framework is useful to understand the mechanobiologic state of a cell based on its morphology, an even more useful application lies in its ability to identify aberrant or atypical cell signaling at the single cell level. In practice, such a tool could be utilized to understand the differentiation and/or invasive potential of cell populations, or even as a screening tool for testing the efficacy of drug treatments to restore a healthy morpho-mechanobiologic relationship in aberrant cells.

## Methods

### Fibrous scaffold fabrication

Fibrous scaffolds were fabricated by electrospinning as previously described^2^. Briefly, 19% w/v poly(ε-caprolactone) was extruded through a needle charged to + 15 kV and collected onto a rotating mandrel (1 kV/cm voltage gradient to grounded mandrel). Both aligned and nonaligned scaffolds were fabricated based on surface speed of the collecting mandrel (aligned: 14 m/s; nonaligned: 2.3 m/s)^2^. Following collection, scaffolds were rehydrated using 30 min steps of progressively weaker ethanol (100%, 70%, 50%, 30%) prior to being allocated to mechanical analysis or cell seeding. Following 2 × 30 min incubations in phosphate buffered saline, scaffolds for cell analyses were functionalized overnight at 37 °C in 20 µg/mL fibronectin.

### Mechanical analysis and modeling

We sought to model the mechanical behavior of fibrous tissue given a fiber organization^22,24^:

2 $$\mathop \smallint \limits_{ - \pi /2}^{\pi /2} R\left( \theta \right) d\theta = 1$$

where *R(θ)* represents the fiber angle distribution for a given angle *θ*. Under the assumption that the stress of the fiber network is equal to stresses on individual fibers that are oriented along each fiber’s axis, the stress can be described by:

3 $${\varvec{S}} = \mathop \smallint \limits_{ - \pi /2}^{\pi /2} R\left( \theta \right) S_{11}^{f} \left( \varepsilon \right) \left[ {{\varvec{N}} \otimes {\varvec{N}}} \right] d\theta$$

where ***S*** is the second Piola Kirchoff tensor. Here, S_11f._ denotes a fiber stress along the axis of the fiber under a given strain, ε. Fiber mechanics models often rely on a non-linear fiber stress term to account for fiber recruitment and reorientation such as:

4 $$dS_{11}^{f} \left( \varepsilon \right) = A e^{B\varepsilon } d\varepsilon$$

where *A* and *B* are terms that govern fiber stiffness and nonlinearity, respectively. To represent this mechanical model, we fabricated aligned and nonaligned, electrospun poly($\varepsilon$-caprolactone) (PCL) scaffolds. While other models use theoretical approaches to account for fiber reorganization, we tracked fiber organization as a function of strain using an in situ stretch device housed within an environmental scanning electron microscope (Fig. S1a, b), and found that *R(θ)* follows a trend of increasing organization as a function of increased strain (Fig. S2). This fiber organization was then input into the fiber kinematics model. Assuming a fiber angle distribution that is dependent on the state of strain provides:

5 $$R\left( {\theta ,\varepsilon } \right) = \frac{1}{{2\pi \sigma \left( \varepsilon \right)^{2} }} e^{{\frac{{ - \left( {\theta - \theta_{0} } \right)^{2} }}{{2\sigma \left( \varepsilon \right)^{2} }}}}$$

6 $$\sigma \left( \varepsilon \right) = \sigma_{0} e^{ - \alpha \varepsilon } + \sigma_{\infty }$$

where the fiber angle distribution, *R(θ)*, is described by a Gaussian distribution and the standard deviation of this distribution, *σ*, is dictated by the state of strain, the original angular spread, *σ*_*0*_, an organization saturation point, *σ*_*∞*_, and a fitting parameter, *α*. Consequently, the resulting stress–strain relationship can be described by (presented earlier in the results section):

7 $$S_{11} = \mathop \smallint \limits_{0}^{{\varepsilon_{11} }} \mathop \smallint \limits_{ - \pi /2}^{\pi /2} R\left( {\theta ,\varepsilon } \right) \cos^{2} \theta A B \exp \left( {B\varepsilon } \right) d\theta d\varepsilon + k_{matrix} \varepsilon_{11}$$

For mechanical analyses, scaffolds were cut into rectangular strips 10 mm wide and 40 mm long. Thickness was measured using a non-contact laser based method. Scaffolds were clamped into an Instron 5848 mechanical testing frame and stretched at a constant rate of 1 mm/min until failure. Stress and strain were calculated based off initial cross sectional area and initial gauge length of 10 mm. For fiber organization analyses, scaffolds were viewed in a scanning electron microscope (FEI Quanta 600 FEG Mark II). Following imaging, fiber organization was evaluated using a fast Fourier transform approach in the directionality plug-in in ImageJ. The resulting fiber angle distribution was fit to a Gaussian curve (Eq. 4) through root-mean-square error minimization using the Excel solver plug-in. Scaffolds were stretched in the microscope to quantify strain-mediated fiber organization, as recently described^2^. These data were then used to determine the values for (Eq. 5) through root mean square error minimization. The resulting model stress was then determined through a numerical integration of 3° steps for R(θ) and 0.016% strain steps. The fitting parameters were determined through root-mean-square error minimization.

### Cell seeding

Fibrous scaffolds were seeded with several cell types (bovine annulus fibrosus cells, bovine mesenchymal stem/stromal cells, murine dermal fibroblasts from E15, E18, or adults), and cell lines of normal and cancerous human mammary epithelium (MCF-10A, MCF-7, and MDA-MB-231). Supplemental Table 1 describes media formulations and isolation techniques for each cell type. Bovine annulus fibrosus cells were obtained from adult caudal discs as recently described^2^ and bovine MSCs were obtained from juvenile femoral bone marrow as previously described^40^. These cell types were expanded through passage 1 prior to seeding on scaffolds for analysis. Murine dermal fibroblasts were cultured from dermal explant cultures^41^ and utilized prior to P4. Human cell lines were obtained from ATCC and were handled and expanded as recommended by the supplier. Facilities were routinely tested for mycoplasma and all tests have been negative. In all cases, cells were seeded onto fibrous scaffold through drop seeding of 5,000 cells in 0.05 mL growth media onto the scaffold. Cells were allowed to attach for 45 min prior to introducing the culture media to the system. No live animals were directly involved in this study.

### Confocal microscopy and feature identification

Cells seeded on fibrous scaffolds were fixed in 10% neutral buffered formalin for 18 min following 24 h of culture. Following fixation, scaffolds were washed twice in PBS followed by 10 min of permeabilization in PBS containing 0.5% Triton X100 supplemented with 0.108 g/mL sucrose. Following 2 washes in PBS, primary antibodies were applied overnight. Primary antibodies used in this study were against YAP/TAZ (mouse anti-YAP/TAZ, Santa Cruz Biotechnology, sc-101199, 1:200 dilution in PBS containing 1% BSA) and α-smooth muscle actin (mouse anti-αSMA, Sigma, A2547, 1:400 dilution in PBS containing 1% BSA). The images for YAP/TAZ for the bAFCs included newly collected images and a subset of re-analyzed images from a recent study, and the images of αSMA staining were images re-analyzed from a recent study^2^. Following two washes in PBS, secondary antibody (AlexaFluor 488, goat anti-mouse, 1:200) and phalloidin (AlexaFluor 546, 1:000 in PBS with 1% BSA) incubation were conducted for 1 h at room temperature. Following PBS washes, scaffolds were mounted with DAPI gold anti-fade. For cases where scaffold autofluorescence hindered nuclear imaging, DRAQ-5 (1:500 dilution) was used to visualize nuclei. Scaffolds were imaged on a Nikon A1R confocal microscope with a 20X objective. For image analysis, z-stacks were imported into ImageJ. Using maximum intensity of collected z-stacks, cells and nuclei were manually identified and segmented. The cell and nuclear shape parameters were determined through internal functions within the software. The parameters collected for each cell were: cell area, cell perimeter, cell major axis length, cell minor axis length, cell bounding length, cell bounding width, cell aspect ratio, cell solidity, cell roundness, cell circularity, cell angle, nuclear area, nuclear aspect ratio, nuclear angle.

### Neural network construction

Three types of neural network models were constructed in this study. All models were constructed using the neural network toolbox in MATLAB (2018). The first model was constructed for dimensional reduction of morphology data by sorting the multidimensional (14 parameter) data into a self-organizing 2 × 2 map determined using 2 neurons. Using this built-in unsupervised learning model, the network was constructed using a competitive learning algorithm on 1043 cells imaged and analyzed as described above. The second model was constructed to predict nuclear/cytoplasmic levels of YAP/TAZ based on the 14 morphology measures. This model was also constructed in MATLAB using the neural network toolbox. Using a 4 neuron hidden layer with sigmoid transfer functions, the model was trained using Bayesian regularization on 75% of the cell data (253 cells) and the model was independently validated using the remaining 25% of cell data (85 cells). The initial model was developed using bovine annulus fibrosus cells seeded on aligned and nonaligned scaffolds that were stretched to either 0% or 9% strain (Fig. 3). This model was then updated by retraining with the annulus fibrosus cells, bovine mesenchymal stem cells, and adult murine dermal fibroblasts (699 cells total, Fig. 4). Finally, this model was updated to include human epithelial cells both prior to- and following initiation of epithelial to mesenchymal transition, using the MCF-10A cell line with and without addition of 10 ng/mL TGFβ_1_. This model was trained on 814 total cells (Fig. 5). In addition to the morphology-only model, another model was constructed that incorporated known biochemical cues into the input data as a 15th input. In this, a contractility agonist (LPA) was assigned a value of 1, control media was assigned 0, and a contractility inhibitor was assigned a value of − 1. This model was developed solely on bAFCs. Visual depictions of predicted states were constructed using Inkscape v0.92 vector graphics software. Images of cell and nucleus were imported and outlined, cytoplasmic levels of YAP/TAZ were recorded on a [0–255] scale and assigned to the cytoplasm, nuclear intensity was then assigned from the predicted YAP/TAZ levels on a [0–255] scale. The final model was constructed to classify cells into two classes (invasive versus non-invasive). Once again using the neural network toolbox in MATLAB, either the morphology, the YAP/TAZ data, or the combination of the morphology and YAP/TAZ data were used to develop the model to classify cells as invasive (MDA-MB-231) or non-invasive (MCF-7). Using random data partitioning of 50% training, 15% validating, and 35% testing, the model was trained through scaled conjugate gradient backpropagation, with a 10 neuron hidden layer. Confusion matrices for the testing set are reported for each of the 3 sets of input data.

### Statistical analyses

For comparisons between model prediction errors for different groups a non-parametric test (Kruskal–Wallis) was used due to unequal variances between groups. Dunn’s post hoc test was used to find differences between groups. Significance was set at p < 0.05. Analyses were conducted using Graphpad Prism 7. Outliers were identified and removed from the initial training set YAP/TAZ values via a Grubb’s test.

## Supplementary Information

Supplementary Information
