# Supplementary material for: Cell morphology and mechanosensing can be decoupled in fibrous microenvironments and identified using artificial neural networks
Source: Sci Rep. 2021 Mar 15;11:5950. doi: 10.1038/s41598-021-85276-5 (PMC7961147; doi:10.1038/s41598-021-85276-5)
Supplement: Supplementary file 1 — Supplementary Information [file 41598_2021_85276_MOESM1_ESM.docx]

**Supplemental Material:**

**Cell morphology and mechanosensing can be decoupled in fibrous microenvironments and identified using artificial neural networks**

Edward D. Bonnevie^1,2^, Beth G. Ashinsky^2,3^, Bassil Dekky^4^, Susan W. Volk^4^, Harvey E. Smith^1,2^, *Robert L. Mauck^1,2,5^

^1^McKay Orthopaedic Research Laboratory, Orthopaedic Surgery, University of Pennsylvania

^2^Translational Musculoskeletal Research Center, CMC VA Medical Center

^3^Department of Biomedical Engineering, Drexel University

^4^Department of Clinical Sciences and Advanced Medicine, School of Veterinary Medicine, University of Pennsylvania

^5^Department of Bioengineering, University of Pennsylvania

***corresponding author:** [**lemauck@pennmedicine.upenn.edu**](mailto:lemauck@pennmedicine.upenn.edu)


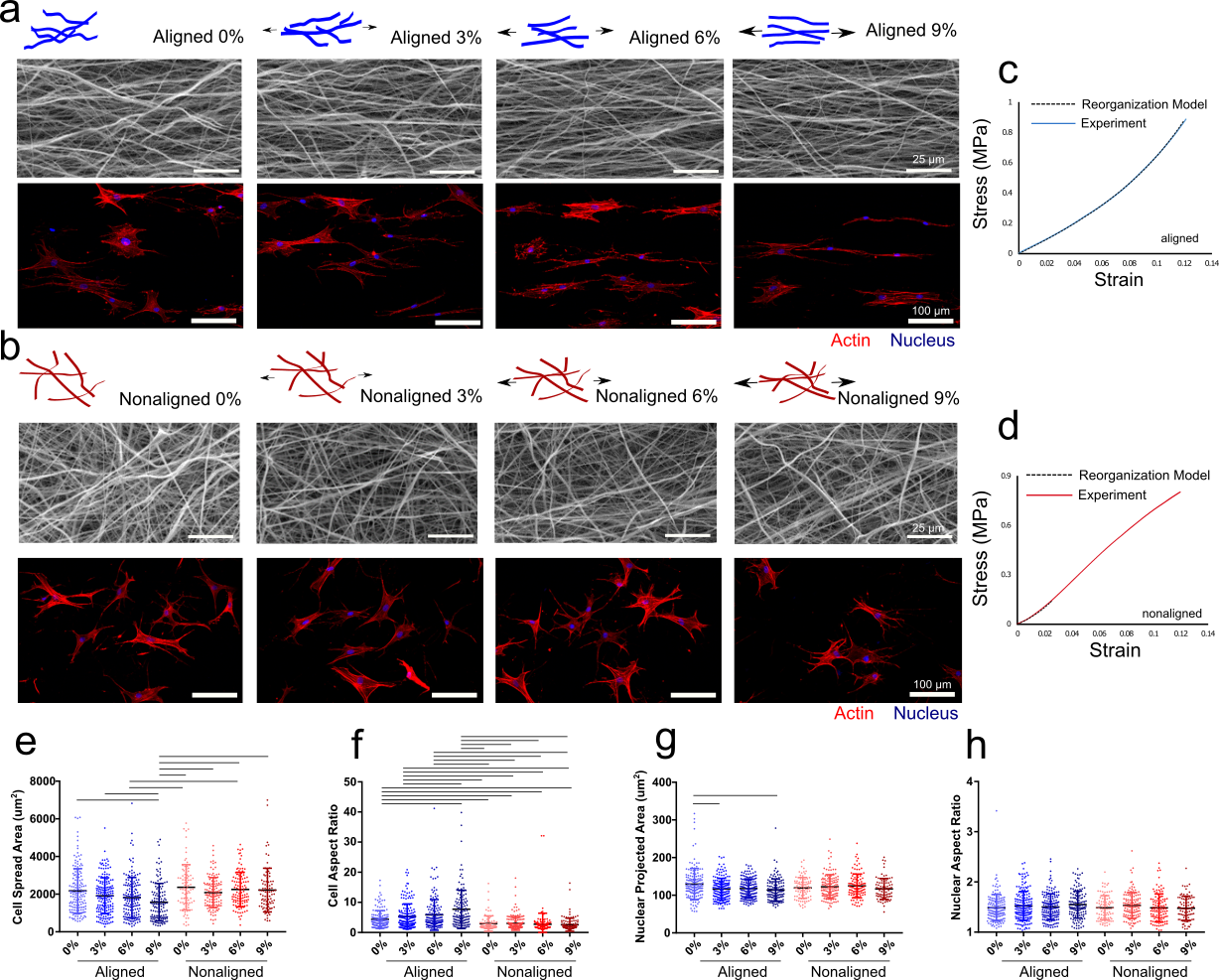


**SFigure 1.** Engineered fiber environments exhibit strain mediated topography and contact guidance. (a) aligned and (b) non-aligned electrospun PCL scaffolds show fiber reorganization as a function of stretch (0% to 9% axial strain) and these topographic cues mediate cell spreading in these fiber environments. Fiber reorganization is consistent with the strain-stiffening behavior of the toe-region in uniaxial tension as predicted by a state variable model for both (c) aligned and (d) non-aligned fiber environments. (e-h) While fiber organization can modulate cell and nuclear shape and size, there is considerable heterogeneity in cell spreading in these fiber environments. Bars indicate p < 0.05 between groups (n = 88 - 176 cells per group).

**
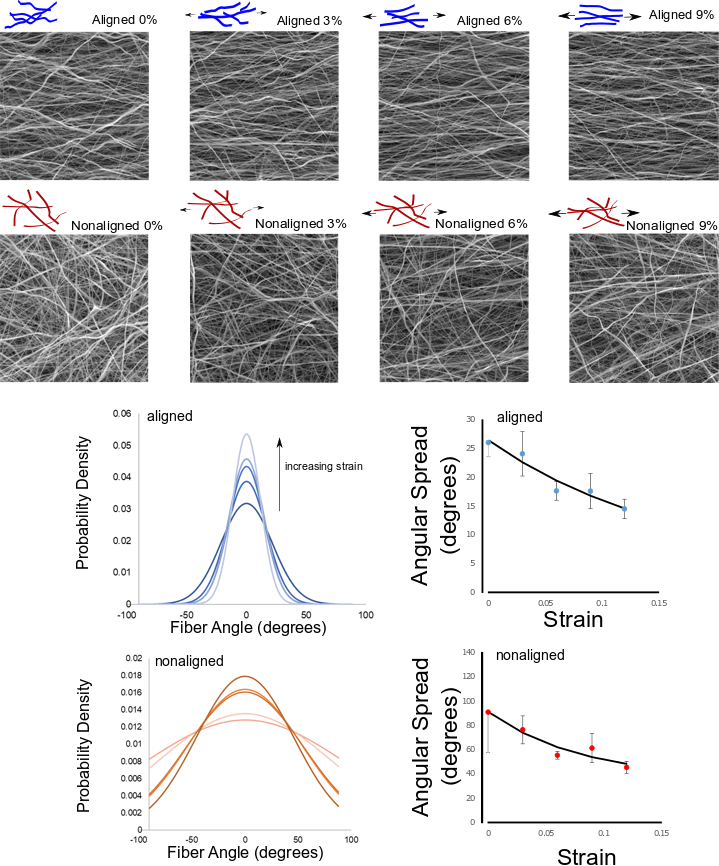
**

**SFigure 2.** Scanning electron microscopy images of stretched scaffolds were assessed for fiber organization through a Fast Fourier Transform-based method. By fitting the fiber angle distributions to a Gaussian curve, the angular spread was calculated as the standard deviation of the curve (n = 3 scaffolds per strain level, averaged of 3 ROIs per scaffold).


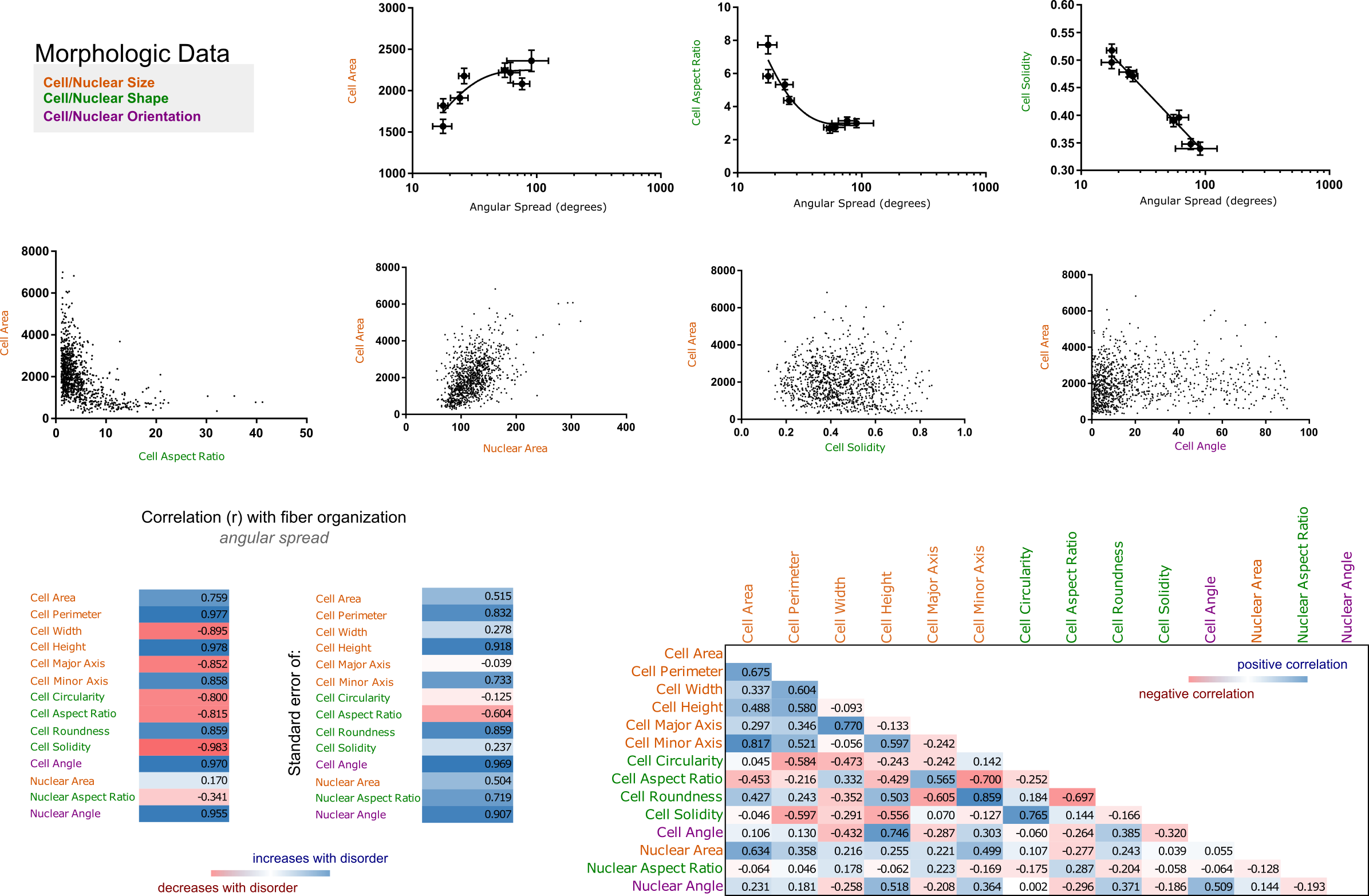


**SFigure 3.** Individual cell morphology data representing cell and nuclear size shape and orientation. This 14-dimensional data consisted of parameters that were closely related to fiber organization, as well as individual cell parameters that were related to one another (n = 1043 cells).


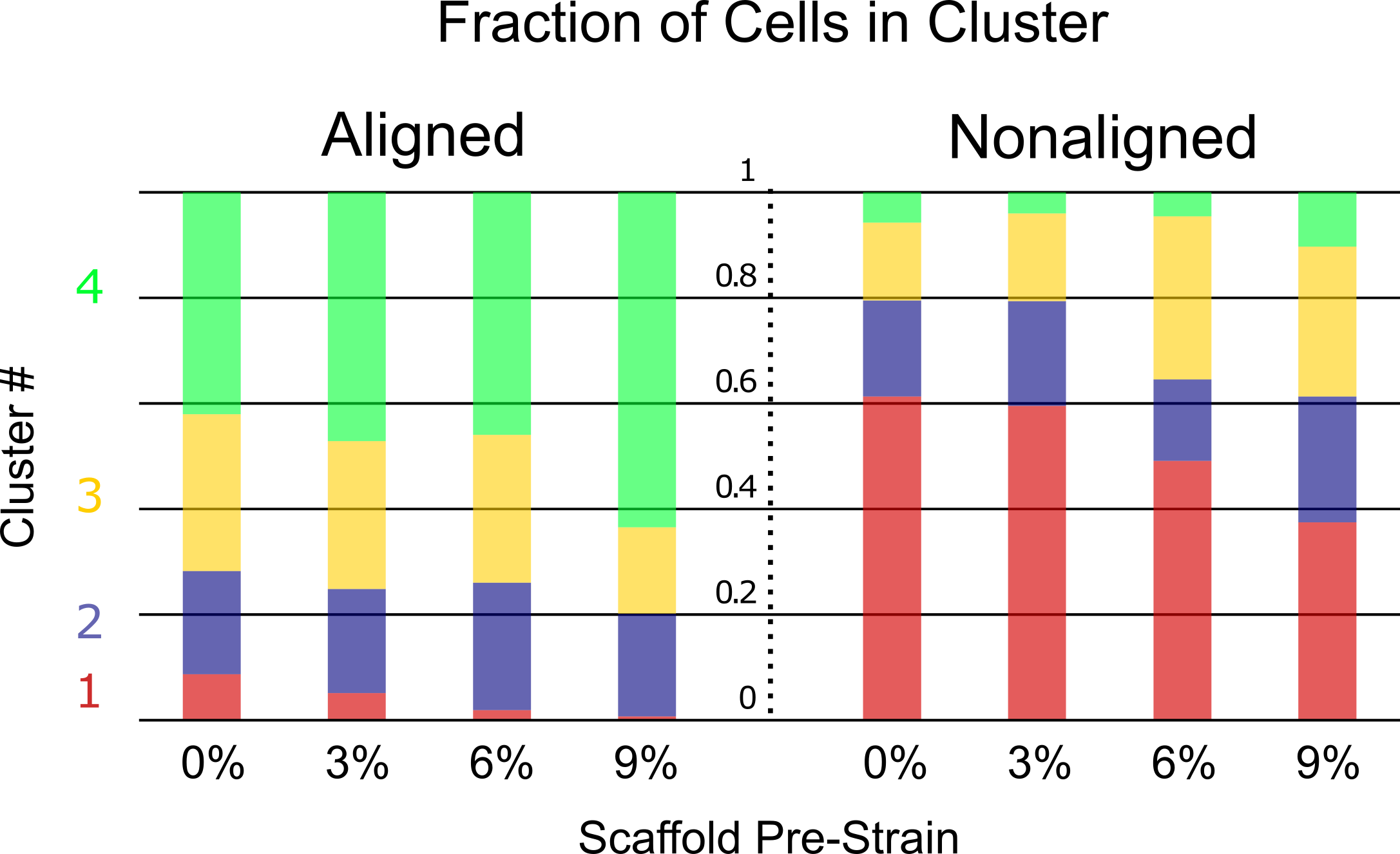


**SFigure 4.** The proportion of cells in the identified clusters depended on scaffold organization (aligned or nonaligned) and the degree of pre-strain mediated fiber reorganization.


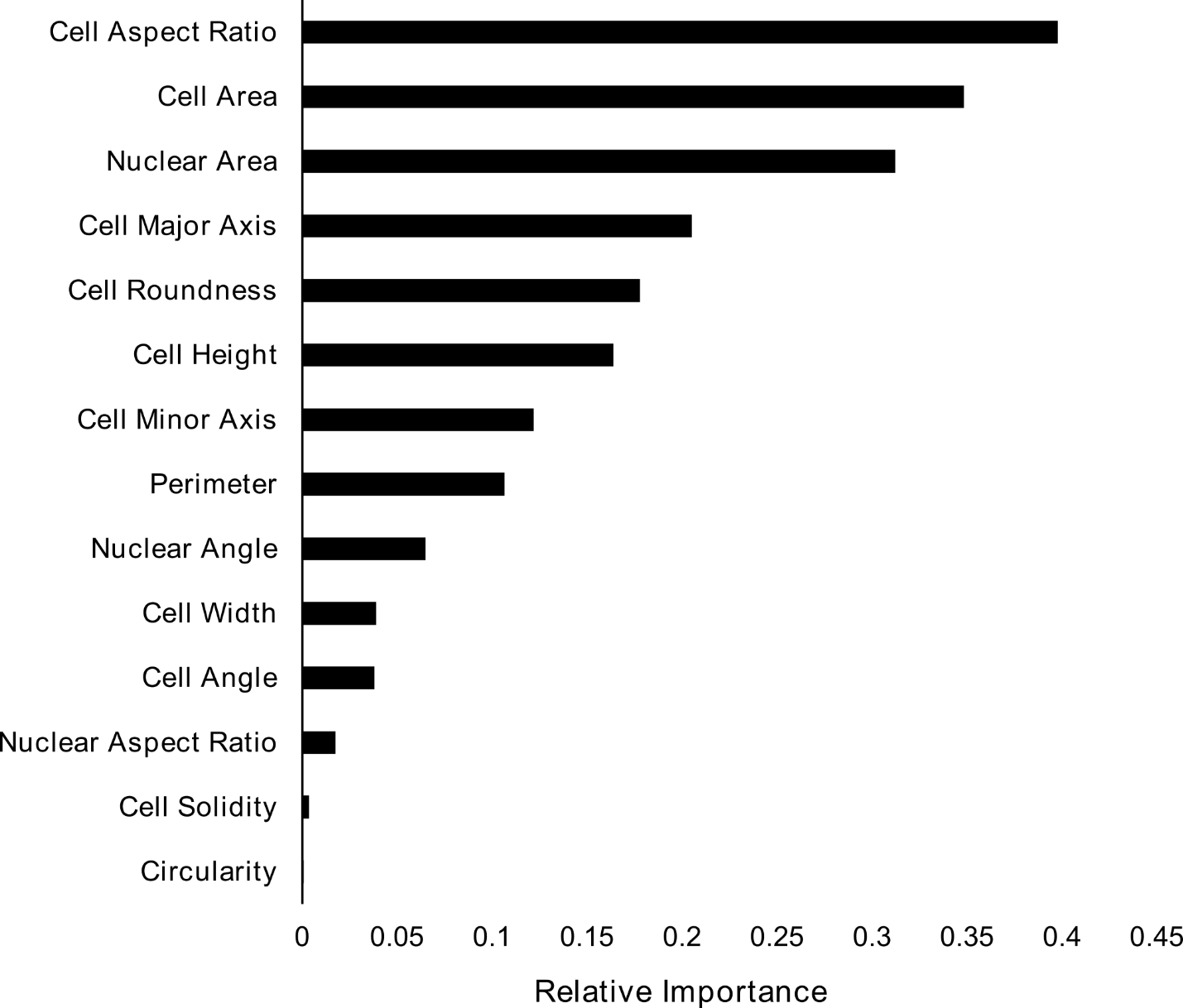


**SFigure 5.** Relative importance of the 14 parameters describing cell and nuclear morphology in the neural network constructed to predict YAP/TAZ signaling.

**
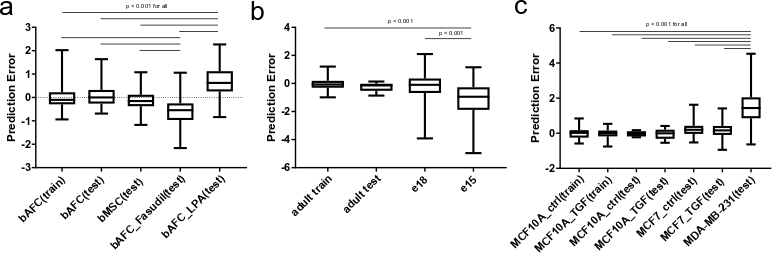
**

**SFigure 6.** (a) Statistical analysis of prediction errors from the morphology only model revealed cells treated with Fasudil or LPA were significantly over or underestimated, respectively. (b) Statistical analysis of prediction errors revealed early embryonic dermal fibroblasts have overestimated YAP/TAZ levels compared to adults and later gestation cells. (c) Statistical analysis of prediction errors revealed invasive cancer cells (MDA-MB-231) have underestimated YAP/TAZ levels compared to other epithelial cells.


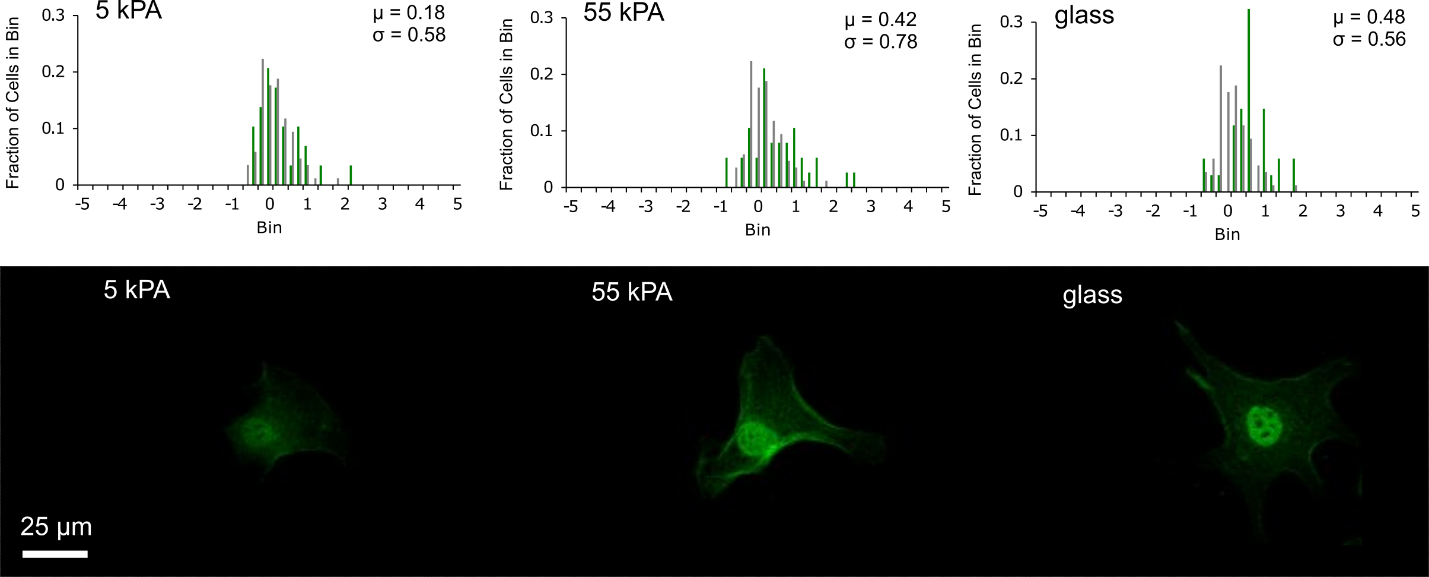


**SFigure 7.** Annulus fibrosus cells seeded onto fibronectin-coated, 2D substrates of different stiffnesses (5 kPa, 55 kPa polyacrylamide and glass) maintained a degree of predictability (n = 29 - 38 cells per group).


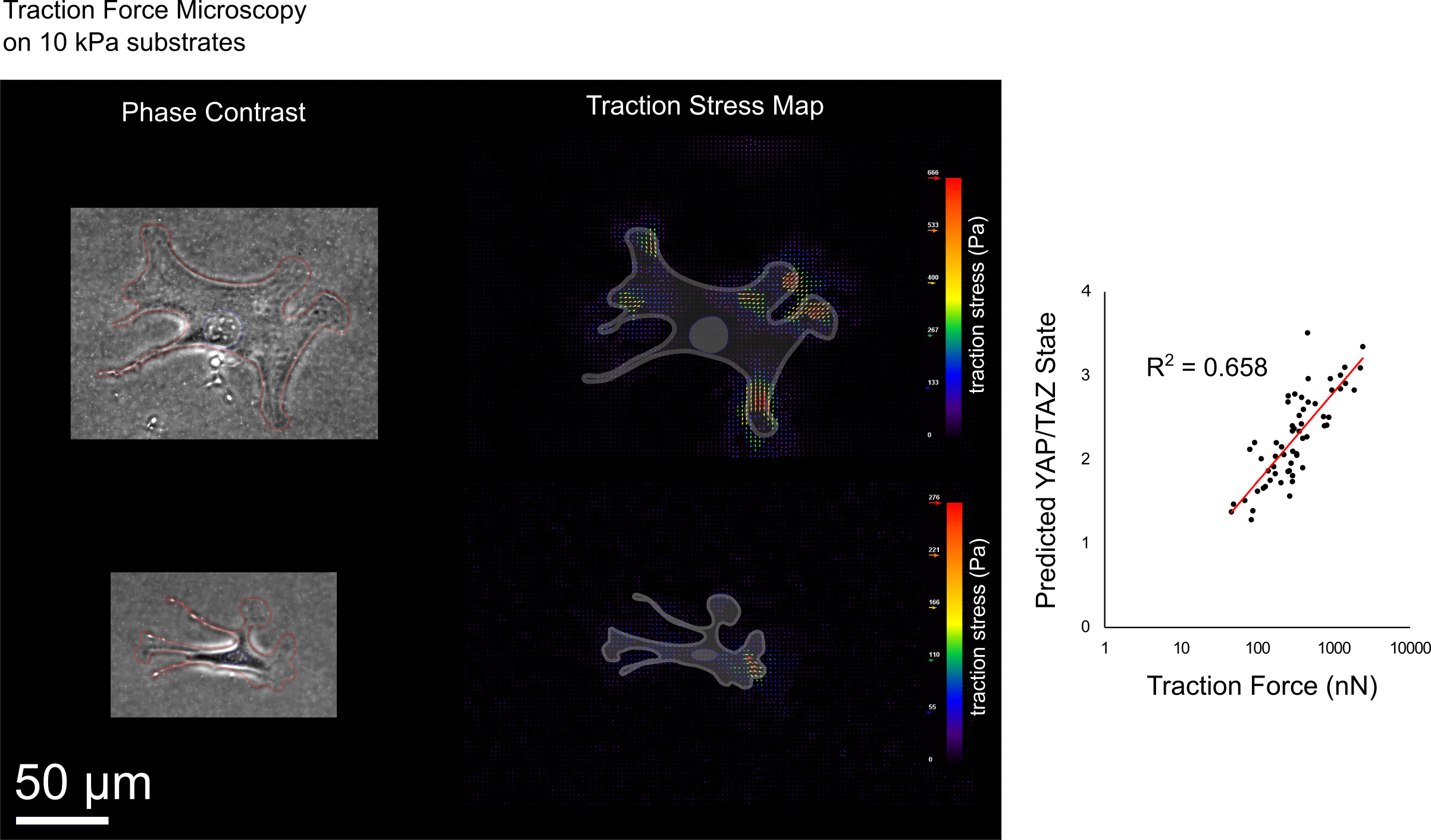


**SFigure 8.** Traction forces for cells on 2D substrates were strongly correlated with predicted YAP/TAZ levels. (n = 60 cells)


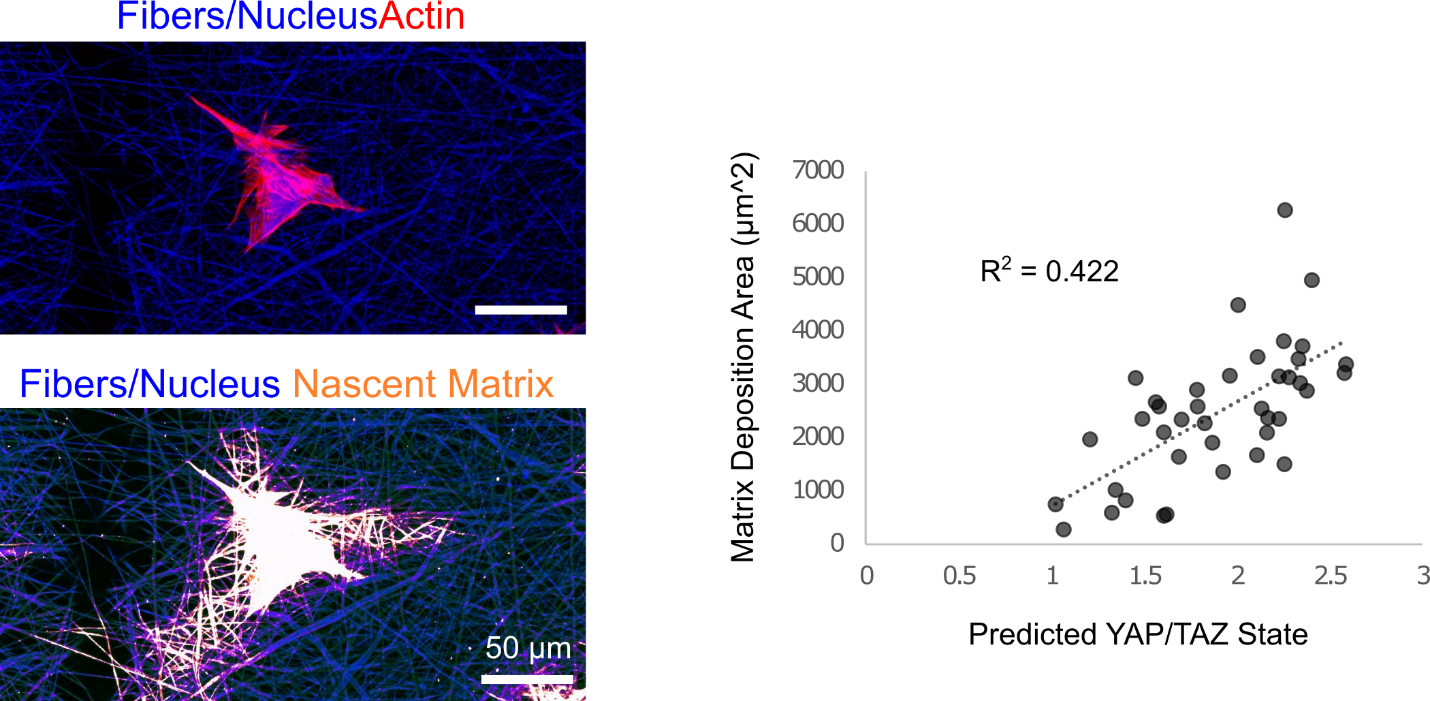


**SFigure 9.** The morphology model was conducted on functional non-canonical amino acid tagging (FUNCAT) data recently reported^1^ and matrix deposition area maintained a significant correlation with predicted YAP/TAZ state (n = 40). (^1^Bonnevie, E. D. *et al.* Aberrant mechanosensing in injured intervertebral discs as a result of boundary-constraint disruption and residual-strain loss. *Nat. Biomed. Eng.* (2019). doi:10.1038/s41551-019-0458-4)


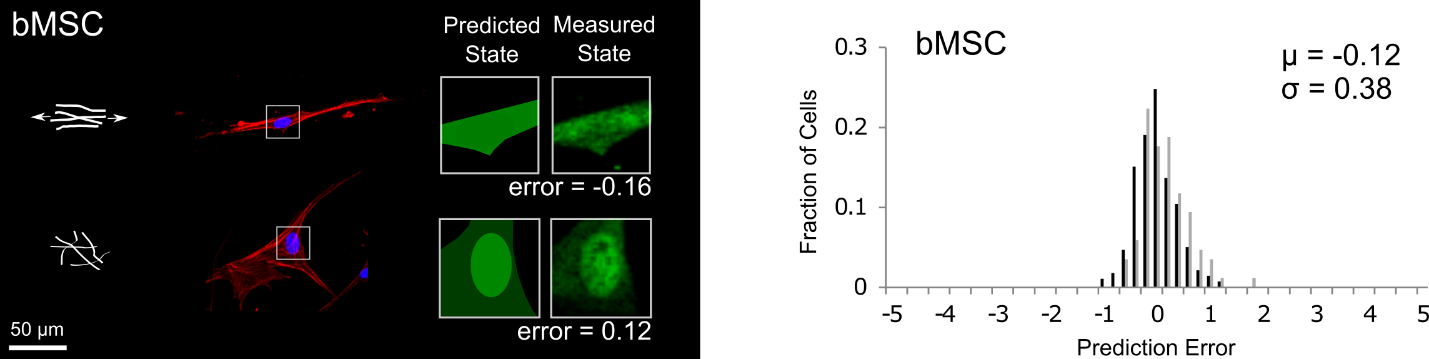
**SFigure 10.** Bovine MSCs were independently tested in morphology-based neural network with accuracy close to that of the bAFCs (n = 278 cells).


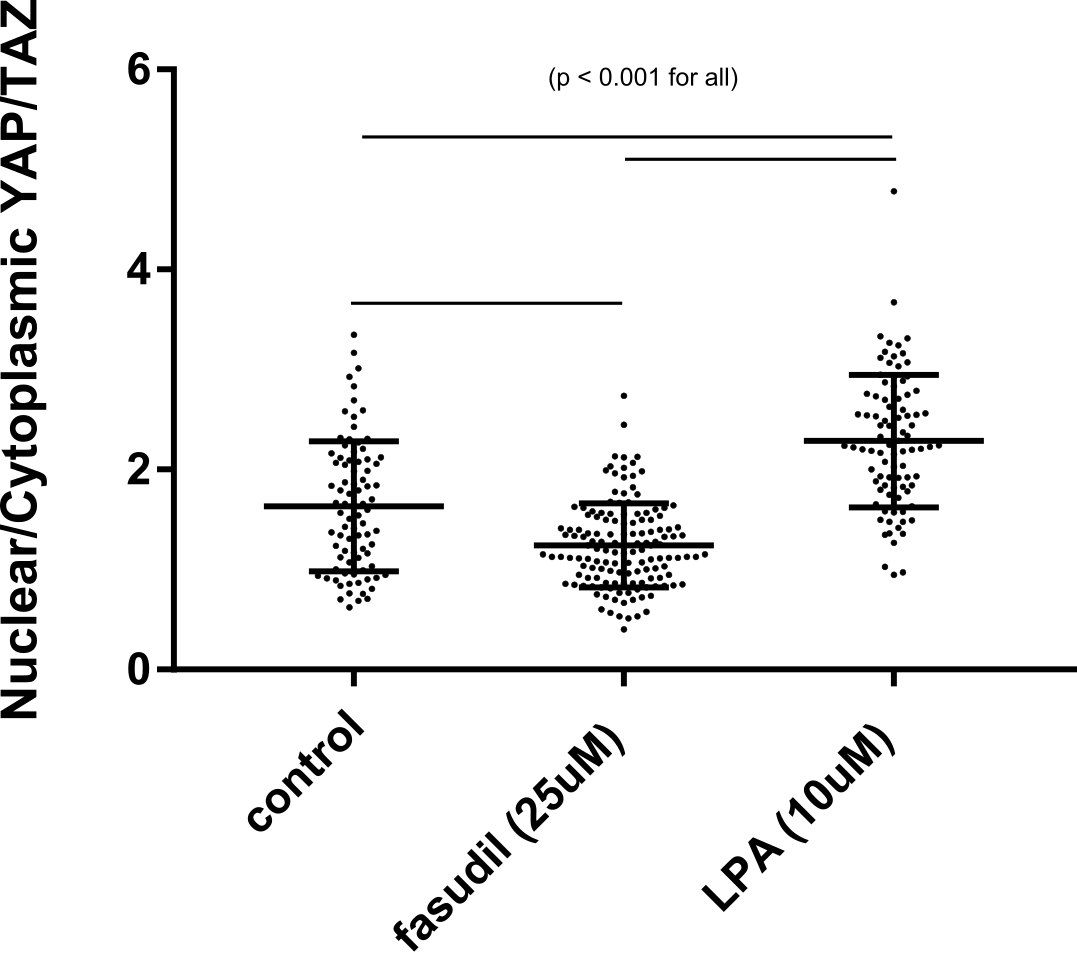


**SFigure 11.** Increasing and decreasing cellular contractility with the ROCK inhibitor fasudil or RhoA agonist LPA modulates nuclear/cytoplasmic levels of YAP/TAZ (n = 85 – 142 cells per group).


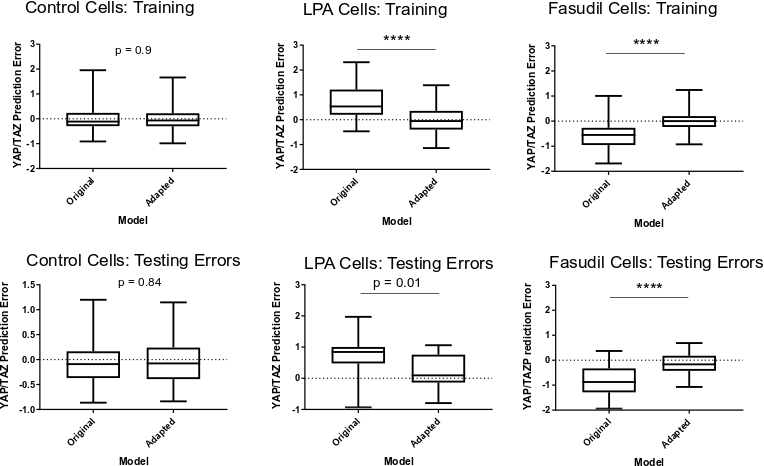


**SFigure 12.** Error graphs for cells used in the training and testing of both the original (morphology only) or adapted model (morphology and biochemical cues) showing enhanced accuracy when biochemical cues are incorporated as input data into a neural network model (n_LPA-train_ = 73, n_LPA-test_ = 16, n_Fasudil-train_ = 112, n_Fasudil-test_ = 30, n_control-train_ = 299, n_control-test_ = 39 cells; **** p <0.0001).


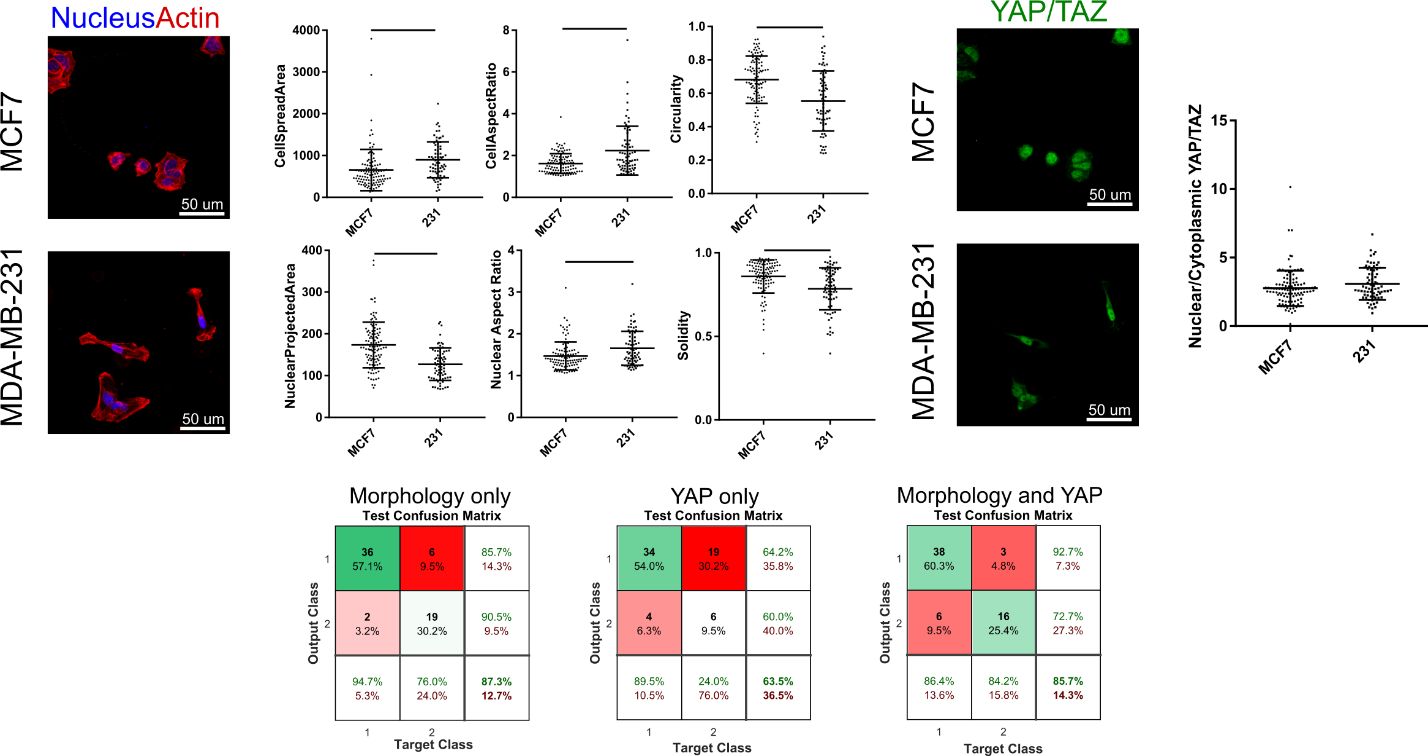


**SFigure 13.** Cancer cells seeded onto glass substrates do not have differences in YAP/TAZ signaling between invasive and non-invasive cancer cells (MDA-MB-231 and MCF-7, respectively). This disconnect relative to their behaviors in fiber environments is highlighted by decreased accuracy in classifying the 2 cell types by a neural network informed by both morphology and mechanobiology. Bars denote p < 0.05. (Target class 1: MDA-MB-231; Target class 2: MCF-7) (bars denote p < 0.05, n_MCF-7_ = 109 and n_MDA-MB-231_ = 72 cells)

| Cell Type | Isolation technique | Growth Media | Analysis Media |
| --- | --- | --- | --- |
| Bovine annulus fibrosus cells | 0.1% collagenase digestion | high glucose DMEM + 10% FBS, +1% AB/AM | high glucose DMEM + 0.1 µM dexamethasone, 50 µg/mL Vitamin C, 40 µg/mL L-proline, 100 µg/mL sodium pyruvate, 1% Ab/AM |
| Bovine MSCs | bone marrow isolate | high glucose DMEM + 10% FBS, +1% AB/AM | high glucose DMEM + 0.1 µM dexamethasone, 50 µg/mL Vitamin C, 40 µg/mL L-proline, 100 µg/mL sodium pyruvate, 1% Ab/AM |
| Mouse e15 dermal fibroblasts | explant crawl out | high glucose DMEM + 10% FBS, +1% AB/AM | high glucose DMEM + 10% FBS, +1% AB/AM |
| Mouse e18 dermal fibroblasts | explant crawl out | high glucose DMEM + 10% FBS, +1% AB/AM | high glucose DMEM + 10% FBS, +1% AB/AM |
| Mouse adult dermal fibroblasts | explant crawl out | high glucose DMEM + 10% FBS, 1% AB/AM, 50 µg/mL Vitamin C | high glucose DMEM + 10% FBS, +1% AB/AM, 50 µg/mL Vitamin C |
| MCF-10A | human cell line | MEGM Mammary Epithelial Cell Growth Medium BulletKit + 100 ng/mL cholera toxin | MEGM Mammary Epithelial Cell Growth Medium BulletKit + 100 ng/mL cholera toxin, +/- 10 ng/mL TGFb1 |
| MCF-7 | human cell line | high glucose DMEM + 10% FBS, +1% AB/AM | high glucose DMEM + 10% FBS, +1% AB/AM, +/- 10 ng/mL TGFb1 |
| MDA-MB-231 | human cell line | high glucose DMEM + 10% FBS, +1% AB/AM | high glucose DMEM + 10% FBS, +1% AB/AM |

**Supplementary Table 1**. Isolation procedures and media formulations for the cell types utilized.
